# Generation of reconstituted hemato-lymphoid murine embryos by placental transplantation into embryos lacking HSCs

**DOI:** 10.1038/s41598-021-83652-9

**Published:** 2021-02-23

**Authors:** Hyojung Jeon, Keigo Asano, Arata Wakimoto, Kaushalya Kulathunga, Mai Thi Nhu Tran, Megumi Nakamura, Tomomasa Yokomizo, Michito Hamada, Satoru Takahashi

**Affiliations:** 1 Department of Anatomy and Embryology, Faculty of Medicine, University of Tsukuba, 1-1-1, Tennodai, Tsukuba, Ibaraki 305-8575 Japan; 2 Laboratory of Stem Cell Therapy, Faculty of Medicine, University of Tsukuba, 1-1-1, Tennodai, Tsukuba, Ibaraki 305-8575 Japan; 3 Ph.D. Program in Human Biology, School of Integrative and Global Majors, University of Tsukuba, 1-1-1, Tennodai, Tsukuba, Ibaraki 305-8575 Japan; 4 Department of Physiology, Faculty of Medicine, Sabaragamuwa University of Sri Lanka, P.O. Box 01, Hidellana, Ratnapura, Sri Lanka; 5 International Research Center for Medical Sciences (IRCMS), Kumamoto University, 2-2-1 Honjo, Chuo-ku, Kumamoto 860-0811 Japan; 6 Laboratory Animal Resource Center, Faculty of Medicine, University of Tsukuba, 1-1-1, Tennodai, Tsukuba, Ibaraki 305-8575 Japan; 7 International Institute for Integrative Sleep Medicine (WPI-IIIS), University of Tsukuba, 1-1-1, Tennodai, Tsukuba, Ibaraki 305-8575 Japan

**Keywords:** Immunological techniques, Immunology, Stem cells

## Abstract

In order to increase the contribution of donor HSC cells, irradiation and DNA alkylating agents have been commonly used as experimental methods to eliminate HSCs for adult mice. But a technique of HSC deletion for mouse embryo for increase contribution of donor cells has not been published. Here, we established for the first time a procedure for placental HSC transplantation into E11.5 *Runx1*-deficient mice mated with *G1-HRD-Runx1* transgenic mice (*Runx1*^*-/-*^::Tg mice) that have no HSCs in the fetal liver. Following the transplantation of fetal liver cells from mice (allogeneic) or rats (xenogeneic), high donor cell chimerism was observed in *Runx1*^*-/-*^::Tg embryos. Furthermore, chimerism analysis and colony assay data showed that donor fetal liver hematopoietic cells contributed to both white blood cells and red blood cells. Moreover, secondary transplantation into adult recipient mice indicated that the HSCs in rescued *Runx1*^*-/-*^::Tg embryos had normal abilities. These results suggest that mice lacking fetal liver HSCs are a powerful tool for hematopoiesis reconstruction during the embryonic stage and can potentially be used in basic research on HSCs or xenograft models.

## Introduction

A mouse model of hematopoietic stem cell (HSC) transplantation requires depleting the HSCs in recipients to empty the niche for donor HSC engraftment. Over the past several decades, total body irradiation^1^, busulfan administration^1^, genetic/antibody-based Kit inactivation^2–4^ and valine starvation^5^ have been established as methods of HSC ablation. Although these methods are effective for the postnatal period, no method has been described for HSC ablation with perfect reconstitution of definitive hematopoiesis during the embryonic stage.

*Runx1/AML1* encodes the DNA-binding protein that binds CBFb/PEBP2b to form the heterodimeric transcription factor complex named core-binding factor (CBF)/polyomavirus enhancer-binding protein 2 (PEBP2)^6^. Runx1 deficiency results in embryonic lethality at E12.5 and a lack of both HSCs and yolk sac-derived erythroid/myeloid progenitors (EMPs) from the hemogenic endothelium^7–13^. Yokomizo et al. generated a *Runx1*^-/-^::*G1-HRD-Runx1* Tg (*Runx1*^-/-^::Tg) mouse using a *G1-HRD-Runx1* transgene with *Runx1* cDNA under the control of the *GATA-1* hematopoietic regulatory domain (*G1-HRD*)^14^. Since GATA-1^+^Runx1^+^ cells in the yolk sac at E7.5 are thought to differentiate into EMPs^15^, the lack of EMPs caused by Runx1 deficiency is partially restored in *Runx1*^-/-^::Tg embryos due to Runx1 expression from the transgene in GATA-1^+^ cells within the yolk sac. Therefore, *Runx1*^-/-^::Tg embryos can survive until E18.5 without HSC-derived definitive hematopoiesis. Since *Runx1* is not expressed in blood vessels or hepatocytes in the fetal liver, the *Runx1*^*-/-*^::Tg fetal liver should be physiologically normal except for a lack of HSCs. Therefore, *Runx1*-deficient fetal livers should be good recipients for donor HSCs in the embryonic stage.

HSC transplantation in the early stages of gestation allows for donor cell engraftment. The advantage of in utero HSC transplantation is the development of donor-specific tolerance, which offers the potential to treat various genetic diseases via the transplantation of healthy donor cells^16, 17^.

Loukogeorgakis et al. demonstrated that HSC injection in the early stages of gestation can be cured by allogeneic transplantation. They show the improved repopulation capacity of transplanted HSCs in an in utero murine model with the continuous delivery of a hydrochloride of CHIR99021 (inhibitor of glycogen synthase kinase-3; GSK-3)^18^.

In 1979, Flechman and Mints demonstrated the hematopoietic engraftment of a c-kit mutant anemic mouse. They displayed complete reconstruction by donor hematopoiesis via the intra-placental injection of allogeneic fetal liver cells at E11 into c-kit mutant embryos and rescued their anemia phenotype^19^.

Takahashi et al. demonstrated immune tolerance induction using intra-chorionic villi injection under ultrasound guidance at E10 in a congenic murine model^20^.

Here, we performed placental hematopoietic cell transplantation into HSCs lacking E11.5 *Runx1*^-/-^::Tg embryos. The results indicated that both donor mouse and donor rat cells contribute to reconstituting the hematopoietic system of a recipient embryo with a higher survival rate. Therefore, this method is useful for analyzing the potential of donor cells in the embryonic stage.

## Methods

### Mice

With respect to donor cell transplantation, cells from Ly5.1 mice with a C57BL/6 background were injected into recipient mice, Ly5.2 *Runx1*^-/-^::Tg mice with a BDF1 background. The generation of *Runx1*^-/-^::Tg mice was previously described^14^. *Runx1*^-/-^::Tg embryos, the recipients of the transplantation, were obtained by mating *Runx1*^+/-^::Tg mice. Genotypes were determined by PCR analysis of tail DNA. For the genotyping, the primers for the Runx1 mutation allele were as follows:

- Runx1a 5′- CAGGTTTGTCGGGCGGAGCGGTAGA-3′,
- Runx1b 5′- CCAAGTCCATCACAGCTGTGCGTT-3′, and
- Runx1c 5′- GCTAAAGCGCATGCTCCAGACTGCCTTG-3’.

The primers for the GATA-1 HRD-Runx1 transgene were as follows:

- RunxF2 5′- CCACAAGTTGCCACCTACCA-3′ and
- RunxR2 5′- CTGGCATCCTGCATCTGACT-3′^1^.

Mice were maintained under specific pathogen-free conditions at the University of Tsukuba Laboratory Animal Resource Center. All experiments were performed in compliance with relevant Japanese and institutional laws and guidelines and were approved by the University of Tsukuba animal ethics committee (authorization number 19–139).

### Donor cell preparation

Fetal liver cells from E14.5 mice (Ly5.1, C57BL/6) or E15.5 rats (“CD(SD)” background) were collected and flushed with PBS using 18 G needles with 1 ml syringes and were then used as donor cells. After they were washed twice with PBS, donor cells were suspended in 3% BSA/PBS.

### Placental transplantation

Pregnant mice (E11.5) were anesthetized, and limbs were fixed with tape on a heating plate. The exposed uterus was placed on a surgical drape and frequently warmed with heated saline at 37 °C during transplantation. An injection pipette was inserted into each placenta. The donor cell suspension (2 × 10^5^ cells/1 μl) was gently injected using a microinjector (FemtoJet, Eppendorf, Hamburg, Germany). After transplantation, the uterus was returned to the maternal cavity, and the incision was closed. During recovery from anesthesia, mice were moved onto a plastic bag containing hot water to avoid hypothermia. Caesarean section was performed 7 days after transplantation (E18.5) for analysis.

### Flow cytometric analysis

The fetal livers and spleens of transplanted embryos (E18.5) were isolated and homogenized using 18 G needles with 1 ml syringes or microscope slides (Matsunami Glass Ind., Ltd., Osaka, Japan). After ACK lysis buffer treatment, cells were stained with the following antibodies. For mouse HSC-transplanted embryos, anti-mouse CD45.1 APC or PE, CD45.2 PE/Cy7 or FITC, CD11b APC/cy7, B220 PE, and CD3 PE (BioLegend, San Diego, CA) were used. For rat HSC-transplanted embryos, anti-mouse CD45.2 FITC or PerCP and anti-rat CD45 PE (BioLegend) were used. Erythroid cells were detected from the fetal livers of rat HSC-transplanted embryos by staining with anti-mouse and anti-rat erythroid cell antibodies (BioLegend). Gallios, CytoFLEX (Beckman Coulter, Brea, CA) and Kaluza v1.3, CytExpert software (Beckman Coulter) as well as BD-LSR (BD Biosciences, San Jose, CA) and FlowJo software (TreeStar, Ashland, OR) were used for the data acquisition and analysis.

### Chimerism analysis

Donor cell chimerism of transplanted mice was calculated using the percentages of donor- and recipient-derived populations obtained via flow cytometric analysis. Calculations were performed as follows:

$${\text{Donor}}\;{\text{cell}}\;{\text{chimerism}}\;\left( \% \right) = \left( {{\text{CD}}45.1^{ + } /\left( {{\text{CD}}45.1^{ + } + {\text{CD}}45.2^{ + } } \right)} \right){\times } 100$$

### Secondary transplantation

For the hematopoietic reconstitution analysis, fetal liver cells were isolated from E18.5 control or rescued *Runx1*^-/-^::Tg, and these cells were then injected into the tail veins of lethally irradiated (7 Gy) 6-week-old female WT (C57BL/6 J;Ly5.2) mice. Survived mice (with greater than 95% chimerism) were used in the further experiments.

### Statistical analysis

Data are reported as the means ± s.e.m. Statistical analysis was performed using R software (version 3.2.2; free download from https://www.r-project.org/). The statistical significance of the differences between the two groups was determined using permuted brunner-munzel test, with *P < 0.05,** *P* < 0.01 indicating statistical significance.

## Results

### ***Placental injection rescued anemia in Runx1***^***-/-***^***::Tg embryos***

**Figure 1:**
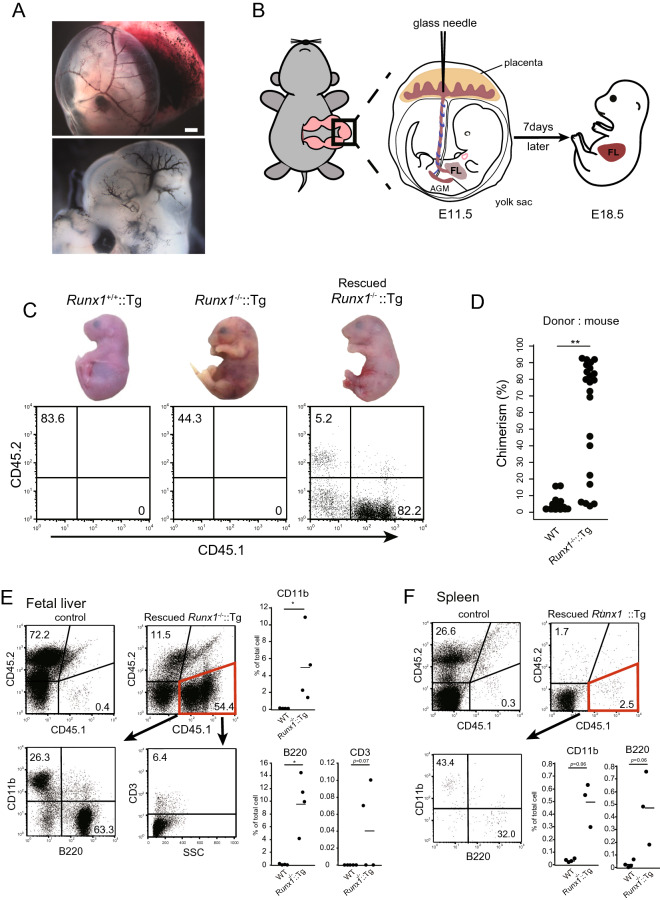
Placental injection rescued anemia in *Runx1*^-/-^::Tg embryos. (**A**) E11.5 embryo after the microinjection of India ink. Ink-filled blood vessels are clearly visible in the yolk sac and heart (upper) and in blood vessels of the brain (lower). Scale bars represent 1 mm. (**B**) A schematic illustration of the experimental outline for assaying transplanted embryos (FL: fetal liver; AGM: aorta gonad mesonephros). The illustration was drawing by using Adobe Illustrator CC (version 25, Adobe Inc.). (**C**) Upper, images of E18.5 *Runx1*^+/+^::Tg (left), *Runx1*-/-::Tg (middle), and rescued *Runx1*-/-::Tg (right) embryos at 30 min after caesarean section. Lower, flow cytometric analysis of the lymphocyte fraction in fetal liver cells for each embryo. Representative pictures and data are shown. (**D**) Plots indicate chimerism of CD45-positive donor cells in the livers of WT (n = 25) and *Runx1*-/-::Tg (n = 23) embryos. Asterisks indicate statistical significance. **P < 0.01. (**E**) CD45.1-positive donor-derived cells in the liver of rescued *Runx1*^-/-^::Tg embryos (E18.5) were analyzed for CD11b (a macrophage marker), B220 (a B cell marker), and CD3 (a T cell marker) by flow cytometry. Data are representative of three independent experiments. Dot plots show percentage of CD11b, B220 and CD3 positive cells among gated donor derived CD45.1 positive cells in fetal liver. Asterisks indicate statistical significance. *P < 0.05; **P < 0.01. (**F**) CD45.1-positive donor-derived cells in the spleen of rescued *Runx1*^-/-^::Tg embryos (E18.5) were analyzed for CD11b and B220. Data are representative of two independent experiments. Dot plots show percentage of CD11b and B220 positive cells among gated donor derived CD45.1 positive cells in fetal spleen. Asterisks indicate statistical significance. *P < 0.05; **P < 0.01.

At the beginning of this study, we attempted to inject donor cells into 232 embryos via an intrahepatic route at E13.5 or E14.5. Although WT embryos survived at E19.5, we never obtained rescued *Runx1*^-/-^::Tg embryos at this stage, suggesting that embryos may be damaged by the injection process (supplemental Table 1). To avoid injuring embryos, placental injection was performed at the early embryonic stage. For the practice experiment, India ink was injected into E11.5 embryos through the placental side of the uterine wall. As shown in Fig. 1A, injected India ink was observed in blood vessels of both the yolk sac and the embryo. Next, E14.5 fetal liver cells from the congenic donor strain C57BL/6-Ly5.1 were injected, followed by closure of the peritoneum and skin of pregnant *Runx1*^+/-^::Tg mice with sutures. E18.5 embryos were obtained seven days after injection (Fig. 1B). Out of 401 embryos injected at E11.5, 267 embryos remained alive until E18.5 (survival rate: 66.6%; supplemental Table 2). The complexion of certain injected *Runx1*^-/-^::Tg embryos was normal, suggesting that hematopoietic reconstitution was successful (Fig. 1C, upper panel). We next examined the chimerism of liver cells via flow cytometry, using anti-CD45.1 and anti-CD45.2 antibodies. As expected, most hematopoietic cells of rescued E18.5 *Runx1*^*-/-*^::Tg embryos were from donor-derived CD45.1 cells (Fig. 1C, lower panel). We obtained 14 *Runx1*^*-/-*^::Tg embryos with more than 70% chimerism from 32 injected *Runx1*^*-/-*^::Tg embryos; no WT embryos exhibited higher chimerism (Fig. 1D). We also used flow cytometric analysis to evaluate whether donor cells contributed to lymphocyte populations and found that in the liver, they could significantly contribute to CD11b-positive myeloid cells, cells in the B220-positive B cell lineage, and cells in the CD3-positive T cell lineage (Fig. 1E). In the spleen, although there were few contributed donor cells, CD11b-positive and B220-positive cells were present (Fig. 1F). Moreover, CD45.1-positive donor cells were detected in both the spleen and thymus (supplemental Fig. 1A, B). B220-positive cells and CD3-positive cells were also detected in the spleen and thymus, respectively (supplemental Fig. 1C, D). The HE staining data indicated that there are no morphological differences between control and *Runx1*^-/-^::Tg embryo (supplemental Fig. 1E, F). These data suggest that donor cells contribute to not only the liver but also the spleen and thymus. Overall, our data indicated that donor hematopoietic cells successfully contributed to the *Runx1*^*-/-*^::Tg hematopoietic system, especially in the fetal liver. Moreover, although the anemic phenotype of the *Runx1*^-/-^::Tg mice was alleviated, rescued *Runx1*^-/-^::Tg mice died 1–2 h after caesarean section. Because Runx1 plays roles in the nervous system^21–23^ and sternum development^24, 25^, the rescued *Runx1*^-/-^::Tg mice may have died due to manifestations associated with these roles.

### ***Secondary transplantation using rescued Runx1***^***-/-***^***::Tg E18.5 fetal liver cells in adult recipient mice***

**Figure 2:**
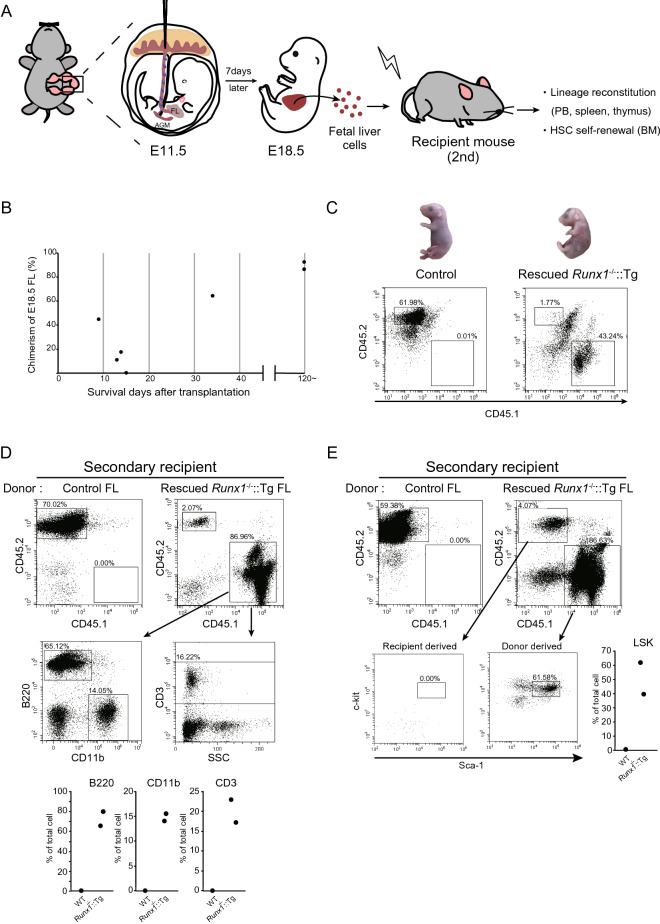
Rescued *Runx1*^-/-^::Tg embryo HSCs retain their potential to sustain hematopoiesis after secondary transplantation to adult recipient mice. (**A**) A schematic diagram of the experimental outline for secondary transplantation. (FL: fetal liver; AGM: aorta gonad mesonephros; PB: peripheral blood; BM: bone marrow). The illustration was drawing by using Adobe Illustrator CC (version 25, Adobe Inc.). (**B**) Plots indicate the survival day of the secondary recipient mouse. The Y axis indicates chimerism on E18.5 rescued *Runx1-/-*::TG. The X axis indicates the days after secondary transplantation. (**C**) Chimerism analysis of donor cells of secondary transplantation recipients. Upper, images of E18.5 *Runx1*^+/-^::Tg (left) and rescued *Runx1*-/-::Tg (right) embryos. Lower, flow cytometric analysis of the donor cell (CD45.1-positive) lymphocyte fraction in fetal liver cells for each embryo. Representative pictures and data plots are shown. (**D**) The peripheral blood analysis of recipient mice after secondary transplantation. CD45.1-positive donor-derived cells were analyzed for B220 (a B cell marker), CD11b (a macrophage marker) and CD3 (a T cell marker). Data are representative of two independent experiments. Dot plots show percentage of CD11b, B220 and CD3 positive cells among gated donor derived CD45.1 positive cells in the peripheral blood. Asterisks indicate statistical significance. *P < 0.05; **P < 0.01. (**E**) Data of the bone marrow cells of recipient mice after secondary transplantation. Lower, flow cytometric analysis for c-kit + and Sca-1 + cells of CD45.1 donor- and CD45.2 recipient-derived lineage-negative cells. Dot plots show percentage of lineage negative, Sca1 positive, c-kit negative (LSK) cells among gated donor derived CD45.1 positive cells in the bone marrow. Asterisks indicate statistical significance. *P < 0.05; **P < 0.01.

Because the rescued *Runx1*^-/-^::Tg embryos died after birth, the self-renewal ability of HSCs in the *Runx1*^-/-^::Tg fetal livers could not be evaluated. To confirm the abilities of HSCs in the fetal liver cells of rescued *Runx1*^-/-^::Tg, we performed secondary transplantation using E18.5 fetal liver cells to X-ray lethally-irradiated recipient adult mice (Fig. 2A). The results indicated that 2 mice with high chimerism (more than 80%) fetal livers survived for at least 6 months whereas almost all of the low chimerism (less than 50%) embryos died approximately 2 weeks after the secondary transplantation (Fig. 2B, C). The flow cytometry data of the bone marrow cells of the surviving mice showed that the CD45.1 donor cells contributed to B220-, CD11b- and CD3-positive cells (Fig. 2D). Moreover, lineage-, c-kit + , and Sca-1 + undifferentiated cells that included HSCs were observed only in the CD45.1 donor-derived cells (Fig. 2E). These data indicate that HSCs in the fetal liver cells of rescued *Runx1*^-/-^::Tg embryos with high chimerism have normal self-renewal and differentiation abilities.

### ***Engraftment of rat hematopoietic cells in Runx1***^***-/-***^***::Tg embryos***

**Figure 3:**
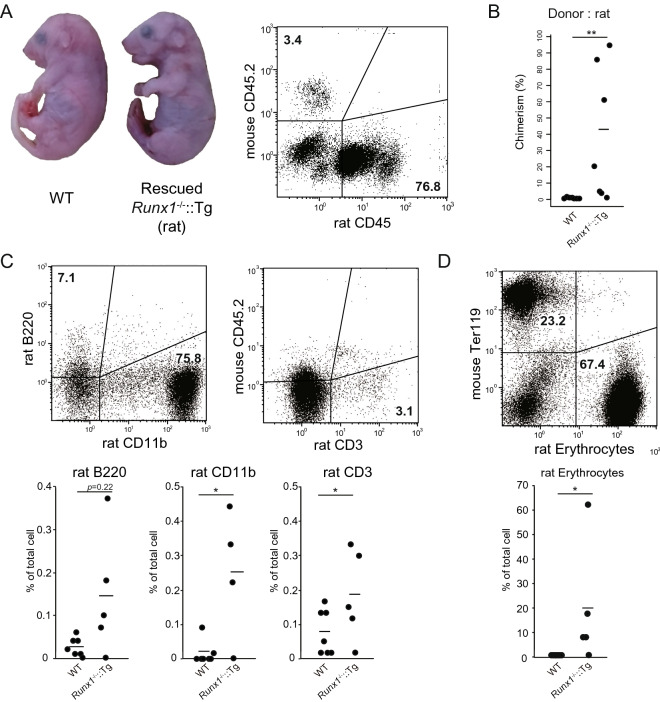
Rat hematopoietic cells contributed to *Runx1*^-/-^::Tg embryo. (**A**) Left, phenotype of *Runx1*^-/-^::Tg embryos rescued by the transplantation of rat hematopoietic cells. Right, flow cytometric analysis of lymphocytes in the livers of *Runx1*^-/-^::Tg embryos with transplanted E15.5 rat fetal liver cells. (**B**) Plots indicate CD45-positive donor rat cells in the livers of chimeric *Runx1*-/-::Tg embryos. Asterisks indicated statistical significance. **P < 0.01. (**C**) Flow cytometric analysis of fetal liver cells from highly chimeric *Runx1*^-/-^::Tg embryos with transplanted rat cells, using rat B220, CD11b and CD3. Lower, data are representative of four independent experiments. Dot plots show percentage of rat CD11b, B220 and CD3 positive cells among gated rat CD45 positive hematopoietic cells in the mouse fetal liver. Asterisks indicate statistical significance. *P < 0.05. (**D**) Flow cytometric analysis of erythrocytes in *Runx1*^-/-^::Tg embryos that received transplanted rat hematopoietic cells. Lower, data are representative of four independent experiments. Dot plots show percentage of rat erythrocytes in the mouse fetal liver. Asterisks indicate statistical significance. *P < 0.05.

In utero hematopoietic cell transplantation is known to induce donor-specific immune tolerance^26^. To examine whether *Runx1*^-/-^::Tg embryos can accept foreign cells from other species, we performed rat hematopoietic cell transplantation into *Runx1*^-/-^::Tg embryos. We collected fetal liver cells from E15.5 rat embryos and injected them into E11.5 embryos via the placenta in pregnant *Ruxn1*^+*/-*^::Tg females; 36 surviving embryos were obtained from 53 injected embryos. Three xenotransplanted *Runx1*^-/-^::Tg embryos from 5 *Runx1*^*-/-*^::Tg animals were obtained from the 36 surviving embryos and showed normal complexion as well as syngeneic transplantation, which indicate successful hematopoietic reconstitution by rat hematopoietic cells (Fig. 3A, left; supplementary Table 2). Flow cytometric analysis of fetal liver cells from E18.5 xenografted *Runx1*^-/-^::Tg embryos showed high engraftment (96% chimerism) of rat-derived CD45-positive cells (Fig. 3A, Right)*.* The two most chimeric embryos were obtained from 5 rat fetal liver reconstituted mice (Fig. 3B). Moreover*,* rat CD11b myeloid cells, rat CD3-positive T cells and rat B220-positive B cells were detected in the livers of *Runx1*^-/-^::Tg embryos with 96% chimerism (Fig. 3C). In particular, rat Ter119-positive erythrocytes were highly represented in *Runx1*^-/-^::Tg embryos (rat: 67%, mouse: 23%; Fig. 3D). Observations in xenotransplanted *Runx1*^-/-^::Tg embryos indicated that engraftment of rat cells in the liver was achieved by placental transplantation.

## Discussion

Yokomizo et al. reported that *Runx1*^-/-^::Tg embryos lacking fetal liver definitive HSCs could survive for up to 18.5 days before birth with the existence of EMPs, which produce secondary hematopoietic erythrocytes and primitive hematopoiesis derived from yolk sacs^14^. In the present study, we successfully injected HSCs into the placenta of E11.5 *Runx1*^-/-^::Tg embryos before the immune system was constructed. Fetuses with more than 90% allogenic and xenogeneic chimerism were obtained without having to apply any HSC deletion methods such as X-ray irradiation or chemotherapeutic drugs. These results indicate that the method of using an HSC-lacking recipient and placenta injection are useful for inducing immuno-tolerance. Moreover, this method can be applied at earlier stages than other in utero hematopoietic cell transplantation methods.

In this study, to obtain *Runx1*^-/-^::Tg, *Runx1*^+/-^::Tg males were mated with *Runx1*^+/-^ females, and the probability of obtaining *Runx1*^-/-^::Tg was 1/8. Also, the fetus's survival rate is 66.6%; therefore, the probability of getting a *Runx1*^-/-^::Tg individual is only around 8% of all implanted fetuses (Supplemental Table 2). Besides, the probability of obtaining an individual with high chimerism is about 50% of *Runx1*^-/-^::Tg (Fig. 1D, Supplemental Table 2). We need to improve this inefficiency in the future by using *Runx1* conditional knockout mice model. For example, if the male is hematopoietic lineage-specific Cre driver (homo allele) and the female is *Runx1*^*f/f*^, the probability of obtaining *Runx1*-deficient embryos can be increased to 50%.

Hamanaka et al. reported the successful generation of mouse-mouse chimera of blood vessels and hematopoietic cells using blastocyst complementation without HSC ablation, however, there have been very less reports of successful chimerism among different species^27^. Previous reports have shown that C57BL6 (B6) mice and its hybrid do not accept rat bone marrow cells^28^. Our results show that more than 90% chimerism was achieved even with rat fetal liver cells in B6 and DBA hybrid F1 mice. This demonstrates that even xenogeneic cells can be successfully engrafted by placental transplantation into an embryo lacking HSCs without pre-transplantation treatment. Since Takahashi et al. reported that immune tolerance was established by placental transplantation into E10.5, our system seems to have established immune tolerance to rats as well^20^. Thus, this model would be useful for xenograft experiments.

In the current study, the transplantation of fetal liver cells containing HSCs into embryos in the mid-developmental stage allowed us to generate a donor-derived hematopoietic system within mice that lacks HSCs.

Tissue stem cells have a limited differentiation potential, and thus we can avoid the contribution of donor cells to the central nervous system and reproductive lineage, which is one of the major concerns with human-animal chimeras produced by conventional pluripotent stem cell injection into blastocysts. This will lower the ethical hurdle for the medical application of human-animal chimeras^29^.

For establishing humanized immune system in mice, highly immunodeficient mice such as NOD/SCID/Il2rg^-/-^ (NOG, NSG) mice and BALB/c-Rag2^-/-^IL2rg^-/-^ (BRG) mice were used^30–32^. These have 30–60% of human CD45 + cells in the peripheral blood and can be maintained for 6–9 months but have not completely reproduced the human physiological state. Also, either radiation or administration of drugs such as busulfan is required to eliminate HSCs present in recipient mice. On the other hand, our model has the advantage that HSCs do not need to be removed by radiation or drugs because definitive hematopoiesis do not occur in the fetal liver^14^. Therefore, transplantation can be performed from around E10, when secondary hematopoiesis begins in the fetal liver. Moreover, because E10 embryo is small, a fewer donor cells are sufficient. Furthermore, since mice born after transplantation during the fetal period form immune tolerance to donor cells, it may be possible to further transplant the same donor cells after they grow into adults to increase their chimerism.

However, in this paper we use systemic *Runx1*-deficient mice for our analysis, which die after birth due to abnormalities in sternal development and motor neuron^14^. In order to improve this problem, we need to use mice that are deficient in *Runx1* only in hematopoietic cells. But we still have the problem of not being able to suppress the innate immune system, even if we can avoid lethality of transplanted mice by using the hematopoietic specific *Runx1* deficient mice. In other words, primary hematopoiesis is normal in *Runx1*^*-/-*^::Tg, suggesting the presence of primitive macrophages. Since clodronate liposome has been successfully administered to remove macrophages and increase transplantation efficiency, future analysis using clodronate liposome injection via placenta will be necessary^33^. Besides, several cytokines (IL-3, TPO, CSF-1, CSF-2, IL-15, etc.) have considerably different amino acid sequences between mouse and human, which is said to be the cause of the lack of viability of human cells^34^. In recent years, MISTRG mice with human M-CSF, human IL-3, human GM-CSF, human TPO, and the transgene of human SIRPa have been developed to create mice with higher chimerism^35^. Also, human IL-6 knock-in *Rag2*^*-/-*^*IL2rg*^*-/-*^ mice successfully express IgG from human B cells, and Balb/c-*Rag2*^*-/-*^*Il2rg*^*-/-*^*Sirpa*^*NOD*^ (BRGS) overexpressing thymic-stromal-cell-derived lymphopoietin (TSLP) has successfully generated human-like lymph nodes in mice^36,37^. The experimental system in which *Runx1* deficient recipient could be replaced by the experimental system in which highly immunodeficient mice such as *Rag2*^*-/-*^ and *Il2rg*^*-/-*^ mice. By improving the fact that *Runx1*-deficient mice do not express human cytokines and still have the innate immune system derived from primary hematopoiesis, it may be possible to create humanized mice with higher chimerism.

For in utero transplantation, injection method via intraplacental, intrahepatic (i.h.), intraperitoneal (i.p.), and intravenous (i.v.) have been established. Recently, Boelig et al. conducted a rigorous comparison of i.h., i.p., and i.v. injection for E14.5 fetuses and showed that i.v. is the most efficient for implantation and is maintained in recipient mice for more than six months^38^. It has been established about intraplacental injection since 1979, and recently a technique for injection into the placental labyrinth of E10 has been reported by using an ultrasound-guided system^19,20^. We have performed the transplantation at E11 in the present study, and the main advantage of intraplacental injection can be conducted earlier than i.v. Also, at embryonic day 9, when the placenta and circulation are established, it seems to be the physical limit of intraplacental injection. Furthermore, as the previous paper has shown, transplantation at this time demonstrates immune tolerance to the donor^20^.

Since the xenograft model using rat HSCs as donors was established in the *Runx1*^-/-^::Tg mice used in this study, it may contribute to the generation of humanized mice using human HSCs. In the future, it will be necessary to create a hematopoietic cell specific *Runx1* deficient mice so that it can be analyzed in adult mice. Also, *Runx1*^-/-^::Tg fetuses can survive until just before birth without definitive hematopoiesis on the fetal liver. The *Runx1*^-/-^::Tg fetuses may be an ideal ‘HSC incubator’ for the physiological conditions of the fetus. In other words, the technique can be used to investigate the differentiation potential of donor fetal-derived HSCs and to analyze the differentiation fate of various blood precursors under more physiological conditions. Thus, this technique may be a tool that can contribute to the field of hematopoietic development in the fetal period.

## Supplementary Information

Supplementary Information 1.
