# Supplementary material for: Generation of reconstituted hemato-lymphoid murine embryos by placental transplantation into embryos lacking HSCs
Source: Sci Rep. 2021 Feb 23;11:4374. doi: 10.1038/s41598-021-83652-9 (PMC7902833; doi:10.1038/s41598-021-83652-9)
Supplement: Supplementary file 1 — Supplementary Information 1. [file 41598_2021_83652_MOESM1_ESM.pdf]

Generation of reconstituted hemato-lymphoid murine embryos by placental transplantation into embryos lacking HSCs

Hyojung Jeon[1,2,*], Keigo Asano[1,*], Arata Wakimoto[1], Kaushalya Kulathunga[1,3,4], Mai Thi Nhu Tran[1], Megumi Nakamura[1], Tomomasa Yokomizo[5], **Michito Hamada**[1,6,†] and **Satoru Takahashi**[1,6,7,†]

[1]Department of Anatomy and Embryology, Faculty of Medicine, University of Tsukuba, 1-1-1, Tennodai, Tsukuba, Ibaraki 305-8575, Japan

[2]Laboratory of Stem Cell Therapy, Faculty of Medicine, University of Tsukuba, 1-1-1, Tennodai, Tsukuba Ibaraki 305-8575, Japan

[3]Ph.D. Program in Human Biology, School of Integrative and Global Majors, University of Tsukuba, 1-1-1, Tennodai, Tsukuba, Ibaraki 305-8575, Japan

[4]Department of Physiology, Faculty of Medicine, Sabaragamuwa University of Sri Lanka, P.O. Box 01, Hidellana, Ratnapura, Sri Lanka

[5]International Research Center for Medical Sciences (IRCMS), Kumamoto University, 2-2-1 Honjo, Chuo-ku, Kumamoto 860-0811, Japan

[6]Laboratory Animal Resource Center, Faculty of Medicine, University of Tsukuba, 1-1-1, Tennodai, Tsukuba, Ibaraki 305-8575, Japan;

[7]International Institute for Integrative Sleep Medicine (WPI-IIIS), University of Tsukuba, 1-1-1, Tennodai, Tsukuba, Ibaraki 305-8575, Japan

*H. J. and K. A. contributed equally to this work.

†Corresponding authors:
Michito Hamada, Ph.D.
Department of Anatomy and Embryology
Faculty of Medicine
University of Tsukuba, 1-1-1 Tennodai, Tsukuba 305-8575, Japan
Phone: +81-298-53-7516
Fax: +81-298-53-6965
E-mail: hamamichi@md.tsukuba.ac.jp
and
Satoru Takahashi, Ph.D.
Department of Anatomy and Embryology
Faculty of Medicine
University of Tsukuba, 1-1-1 Tennodai, Tsukuba 305-8575, Japan
Phone: +81-298-53-7516
Fax: +81-298-53-6965
E-mail: satoruta@md.tsukuba.ac.jp

Abstract

Supplemental Table 1

Number of transplanted embryos and surviving embryos

Mouse	Injected Embryos (E13.5-E14.5)	Surviving embryos (E18.5)	*Runx1*$^{-/-}$::Tg embryo (E18.5)
1	9	3	0
2	2	1	0
3	6	5	0
4	11	1	0
5	9	4	0
6	8	3	0
7	9	6	0
8	8	6	0
9	8	3	0
10	6	5	0
11	7	4	0

The numbers of embryos in the intrahepatic injection are indicated. We did not obtain *Runx1*$^{-/-}$::Tg embryos with intrahepatic transplantation.

Supplemental Table 2

Total number of transplanted embryos.

**Donor**	**Injected embryos (E11.5)**	**Surviving embryos (E18.5)**	***Runx1*$^{-/-}$::Tg embryos**	***Runx1*$^{-/-}$::Tg embryos with high chimerism**
Mouse	401	267	32	14
Rat	53	36	5	2

The numbers of embryos used for both syngeneic and xenogeneic transplantation are indicated. *Runx1*$^{-/-}$::Tg embryos with high donor chimerism contained more than 70% chimeric CD45-positive donor cells in the liver.

Supplimental Fig.1

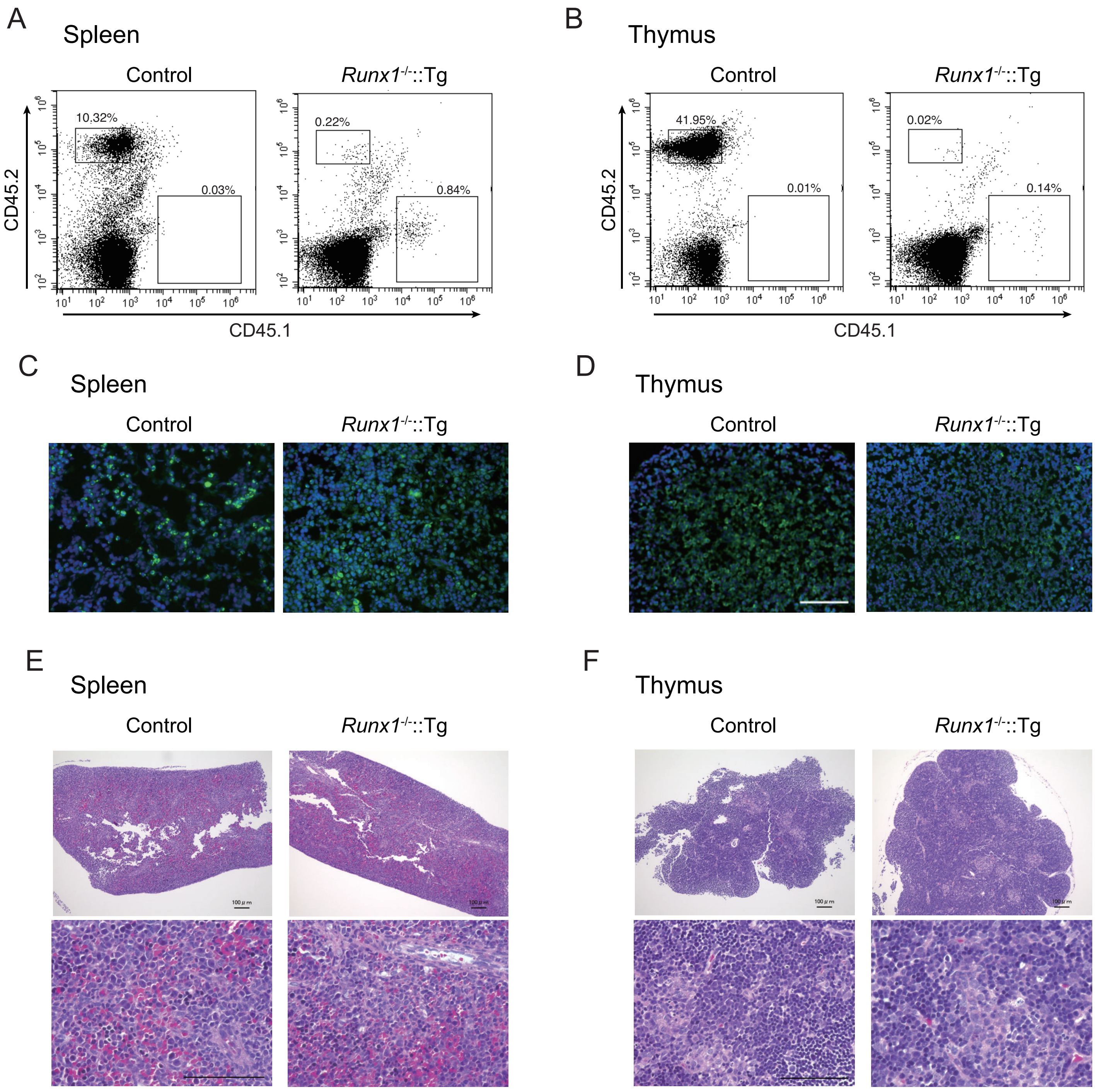

Supplemental Figure 1
(A)Flow cytometric analysis of the lymphocyte fraction of spleen cells from control and *Runx1*$^{-/-}$*::Tg* E18.5 embryos. (B) Flow cytometric analysis of the lymphocyte fraction of thymus cells from control and *Runx1*$^{-/-}$*::Tg* E18.5 embryos. (C) Immunostaining using anti-B220 for control and *Runx1*$^{-/-}$*::Tg* spleens of E18.5 embryo. (D) Immunostaining using anti-CD3 for control and *Runx1*$^{-/-}$*::Tg* thymus of E18.5 embryos (scale bar=100 μm). (E) HE staining of control and *Runx1*$^{-/-}$::Tg spleens of E18.5 embryos. (F) HE staining of control and *Runx1*$^{-/-}$*::Tg* thymus of E18.5 embryos.
